# Rapid Detection of Escherichia coli O157: H7 by Fluorescent Amplification–Based Specific Hybridization (FLASH) PCR

**Published:** 2012-09-30

**Authors:** F Khatami, M Heidari, M Khatami

**Affiliations:** 1 Department of Biology, Science School, Ferdowsi University, Mashhad, Iran; 2 Department of Biology, Science School, Yazd University, Yazd, Iran

**Keywords:** E.coli O157:H7, Stx-1 gene, Detection, FLASH-PCR

## Abstract

**Background:**

Escherichia coli O157:H7 is an enteric pathogen which can be frequently found asymptomatically in ruminant mammals, but can cause diseases from mild diarrhea to hemolytic uremic syndrome in humans.

**Methods:**

We developed fluorescent amplification-based specific hybridization (FLASH–PCR) assay to detect the Stx-encoding gene Stx-1 of E. coli O157:H7.

**Result:**

PCR product of 336 bp was successfully amplified in a FLASH–PCR.

**Conclusion:**

As rapid detection and confirmation of the presence of E. coli O157:H7 are of importance for the medical, food, and water industries, FLASH-PCR is one of selective methods for detection of E. coli O157:H7.

## Introduction

Escherichia coli O157: H7 is an enteric pathogen which can be frequently found asymptomatically in ruminant mammals, but can cause diseases from mild diarrhea to hemolytic uremic syndrome in humans. A small percentage of healthy cattle may normally harbor the organism and are considered to be the primary reservoir of E. coli O157: H7 associated with human disease, as a result of carcasses and hides becoming contaminated by feces during processing (1-3). Food products from other ruminants such as sheep and deer have also been found to be contaminated after processing (4). Potential food sources other than ground meet include unpasteurized juices, milk, dressings and foods such as vegetables that could be contaminated by manure, contaminated water or an infected food handler (2,5). In addition, another major reservoir of disease-causing E. coli O157:H7 can be water contaminated with feces, such as surface water runoff, irrigation water, insufficiently chlorinated municipal water, and swimming water (pools, lakes, beaches) (5).

Molecular approaches for bacterial detection avoid the need for culture and can be designed to be specific. Many molecular-based E. coli assays have been developed. Several polymerase chain reaction (PCR) (6-8), reverse transcriptase PCR (RT-PCR) (9,10), and real-time PCR (11,12), tests have been developed to detect E. coliO157:H7. However, no test used currently detects the eae gene and make it possible to obtain confirmation of negative or positive test results in less than 12 h from the receipt of a sample.

One of the simplest approaches to rapid and sensitive diagnosis and identification of pathogens using DNA technologies is a modification of PCR with the detection of fluorescence during the amplification (real-time PCR) or after its termination (PCR–FLASH) (13). The technique of fluorescent amplification-based specific hybridization (FLASH–PCR), based on determining PCR results by fluorescence intensity, was developed to improve the cost-efficiency of diagnostic laboratories by employing original economical high quality equipment and to eliminate the risk of working zone contamination (13,14).

The aim of the present study was to identification of E. coli O157:H7 via the detection of Stx-encoding gene Stx-1 using FLASH-PCR.

## Materials and Methods

### Bacterial strains

Escherichia coli O157:H7 (ATCC 10798^TM^), Escherichia coli C str. (ATCC 8739), Escherichia coli (Migula) (ATCC 4351) and Escherichia coli (ATCC 25922^TM^) were originally obtained from American Type Culture Collection (ATCC), USA.

### Oligonucleotide primers and Probe design

Primers specific for a conserved region situated within the *E.coli Stx-1* gene were selected (Accession no. AB083044.1). Sequence alignment was performed using Mega 4 (alignment was done using Clustal W, and phylogenetic trees were constructed by neighbor joining) (15,16), and BLAST software (NCBI). Primers and fluorescently labeled probes were designed by primer design software (Primer Premier 5.0; Premier Biosoft Inc., Canada), and their secondary structure was examined with Gene Runner version 3.05 (Hastings Software Inc. Hastings, NY, USA, http://www.generunner.com). All oligonucleotides were synthesized by BIORON Company (Germany).

The fluorescent reporter dye at the 5′ termini of the probe was 6-carboxyfluorescein (FAM), and a quencher of fluorescent was BHQ1 at the 3′ termini. A BLAST search of the GenBank database demonstrates a high predicted specificity, with the cross-reacting bacteria Escherichia coli C str. and Escherichia coli (Migula). To determine the analytical sensitivity of this assay, bacterial DNA for testing was prepared by using the Genomic DNA isolation Kit (GeNetBio, Korea) according to the manufacturer’s instructions. The DNA concentration was determined by measuring the optical density at 260 nm with the GeneQuant spectrophotometer (Pharmacia, Piscataway,N.J.). The DNA concentration was 3 (0.3) mg/ml. Serial dilutions of the extract were tested, and the described assay was able to detect 10 fg of DNA.

### DNA Extraction and FLASH-PCR

DNA extracts were prepared from cultured organisms by using the Genomic DNA isolation Kit (GeNetBio) according to the manufacturer’s instructions.

PCR was performed as suggested by standard manuals (17,18). A 25 μl volumes containing 2.5 μl of 10× PCR buffer (750 mM Tris–HCl, pH 8.8, 200 mM ammonium sulfate, 0.1% Tween-20), 1 mM each of the four deoxynucleoside triphosphates, 3 mM MgCl2, 1 μl of template DNA, 12.5 pM of each primer, 25 pM probe and 2.5 U of Taq DNA polymerase (BIORON, Germany).

The PCR amplifications were performed in a thermal cycler (DNA Technology,Russia) using the following cycling conditions: an initial denaturation at 94°C for 5 min and 30 cycles, with 1 cycle consisting of 30 s at 94°C, 50 s at 58°C for detection of Stx-1, 40 s at 70°C, followed by a final step 2 min at 70 °C, 1 min at 93°C, 1 min at 40°C, and 10 min at 20°C.

FLASH analysis of PCR results was performed using a PCR-detector (DNA technology).

### Electrophoresis

PCR products were separated by electrophoresis in 1% agarose gels in TAE (40 mM Tris–HCl, 20 mM acetic acid, 1 mM EDTA) containing 0.5 μg/ml ethidium bromide and visualized using an Uvitec transilluminator (Gel Imager 2, Russia). The molecular weights were estimated using a Gene Ruler marker (DIALAT Ltd., Russia).

## Results

Species-specific forward (EC-F) and reverse (EC-R) primers generated a PCR product of 336 bp (Table 1).

**Table 1:** Primers and probe for detection of E. coli O157:H7.

Target gene	PCR Product Length	Primer & Probe Seq.	ATCC	Cross-reaction
Stx-1	336 bp	F: 5´-GCGATGTTACGGTTTGTTACT-3´ R: 5´-ACGGACTCTTCCATCTGCC-3´	10798^TM^	1- Escherichia coli C str. ATCC 8739 2-Escherichia coli (Migula) ATCC 4351 3- Escherichia coli ATCC 25922™
		Probe: 5´-FAM -ccctcgcttgccagaatggcatctgatggcgaggg-BHQ1-3´		

**Fig.1:**
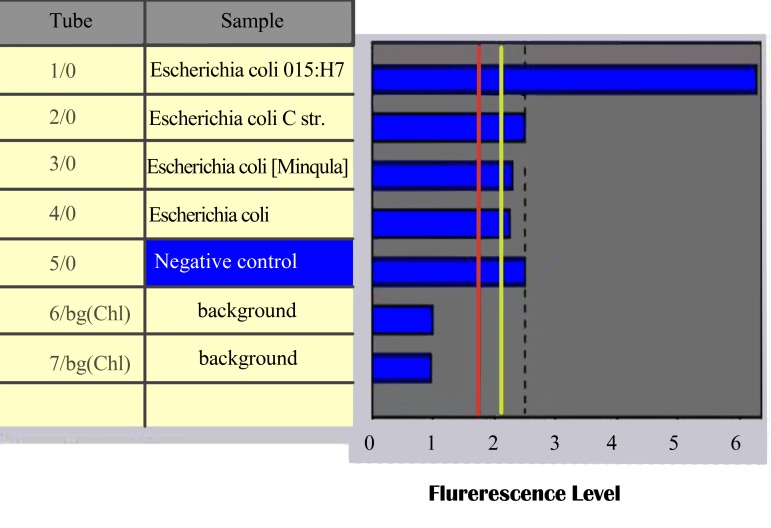
The results of samples by FLASH analysis.

The results of testing the samples using the FLASH format are expressed in (Fig. 1).

**Fig.2:**
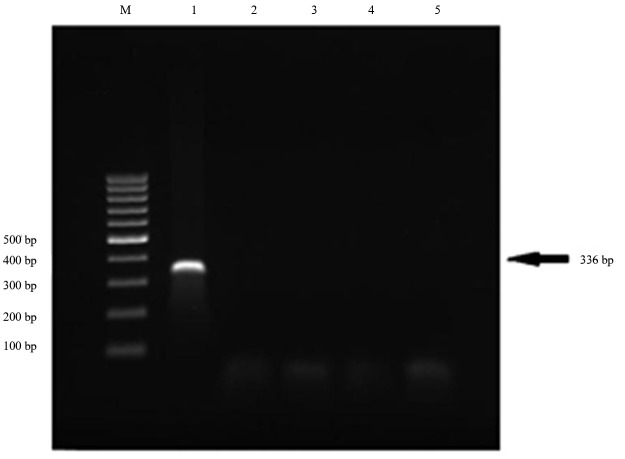
The results of samples by gel electrophoresis. M. Marker, lane 1. E. coli O157:H7, 2. E.coli C. str, 3. E.coli (Migula), 4. E.coli, 5. Negative Control.

A gel electrophoretic analysis of the test system for E.coli (Fig. 2) showed that, with the use of primers strictly specific for this pathogen, only the E.coli O157:H7 is clearly identified (lane 2), whereas the amplification with the DNAs of Escherichia coli C str. and Escherichia coli (Migula) give no amplicons (lanes 1 & 4).

These results showed that during the elongation process in E.coli O157:H7 tube, DNA polymerase destroys the probe due to its 5′-exonuclease activity. This leads to the separation of the fluorophor and quencher and the enhanced fluorescence level.

## Discussion

FLASH –PCR provides a useful approach for the detection of pathogens in environmental and other samples. Numerous researchers have studied and diagnosed *E. coli* O157: H7 using PCR on various samples, such as fecal samples from infected persons or contaminated food samples (raw milk or meat) suspected as causative of outbreak.(19, 20) The use of a single pair of primers specific to target genes that are characteristics of *E. coli* O157:H7, eg, vt eaeA EHEChlyA or uidA, is unable to give an unambiguous positive result as there are a variety of other gram-negative organisms possessing the same target genes of *E. coli* O157:H7 (21, 22).

The visualization of the *E.coli* O157:H7 PCR product by electrophoresis is a laborious and time-consuming process, which inevitably leads upon large-scale screenings to the contamination by amplification products. In order to simplify and accelerate the procedure and avoid the problems associated with possible contamination, we modified the analysis of the results of PCR amplification and transferred it into the FLASH format.

The fluorescence signal for FLASH-PCR of *E. coli* O157:H7 is 6.40 and the maximum fluorescence signal for FLASH-PCR of other related species is 2.50 (Fig 1). These results indicate that these primers and probe is specifics for *E. coli* O157:H7. This modified FLASH-PCR protocol has a potential to be used for rapid, sensitive and specific method in the specific detection of *E. coli* O157:H7.
